# Exploring the Potential of Plant Cytokinins Against Common Human Pathogens: In Vitro Assessment and In Silico Insights

**DOI:** 10.3390/plants14121749

**Published:** 2025-06-07

**Authors:** Jelena Lazarević, Aleksandar Veselinović, Marija Stojiljković, Miloš Petrović, Pierangela Ciuffreda, Enzo Santaniello

**Affiliations:** 1 Department of Chemistry, Faculty of Medicine, University of Niš, Bulevar Dr Zorana Đinđića 81, 18000 Niš, Serbia; aveselinovic@medfak.ni.ac.rs; 2 Veterinary Specialistic Institute Niš, Dimitrija Tucovića 175, 18106 Niš, Serbia; marijavsinis@gmail.com (M.S.); milosvsinis@gmail.com (M.P.); 3 Dipartimento di Scienze Biomediche e Cliniche “L. Sacco”, Universita degli Studi di Milano, 20157 Milano, Italy; pierangela.ciuffreda@unimi.it; 4 Faculty of Medicine, University of Milano, 20122 Milano, Italy; enzo.santaniello@unimi.it

**Keywords:** natural cytokinins (bases and ribosides), in vitro antimicrobial activity, anticandidal agents, molecular docking studies, kinetin and kinetin riboside

## Abstract

Cytokinins, plant hormones derived from adenine, are best known for regulating growth and stress responses in plants. Recent findings suggest they may also influence microbial viability, yet their direct antimicrobial potential remains underexplored. This study evaluates the antimicrobial activities of four natural cytokinins (iPA, B, K, and *p*-T) and their N^9^-ribosides (iPAR, BR, KR, and *p*-TR) against selected human pathogens. Using the broth microdilution method, we assessed their effects on Gram-positive and Gram-negative bacteria, as well as fungal strains. While Gram-negative species showed no susceptibility, all tested compounds exhibited bacteriostatic activity against *Bacillus subtilis* and *Enterococcus faecalis*. Most notably, kinetin (K) and kinetin riboside (KR) displayed strong antifungal activity against *Candida albicans*, with MIC values comparable to the reference drug nystatin. Molecular docking studies supported these findings by showing that K and KR form favorable interactions with two validated antifungal targets in *Candida albicans*: secreted aspartic proteinase 3 (SAP3) and dihydrofolate reductase (DHFR). This is, to our knowledge, the first report linking natural cytokinins to direct antifungal action against *C. albicans* supported by in silico evidence. These findings highlight the potential of K and KR as promising leads for the development of cytokinin-based antifungal agents.

## 1. Introduction

Influencing numerous aspects of growth, development and physiology, N^6^-substituted adenines and the corresponding nucleosides represent a significant group of phytohormones called cytokinins. As chemical regulators involved in diverse biochemical processes in plants, cytokinins play major roles in the regulation of various processes associated with active growth, metabolism, and plant development [1]. Also known to play a role in the synthesis and maintenance of chlorophyll, cytokinins are known to influence chloroplast development and metabolism. As such, cytokinins have long been known to delay senescence [2] and to impact plant nutrient translocation by converting source tissues into active sinks [1]. Finally, they are known to play a role in integrating diverse stress responses and environmental factors, including the defense response against pathogens [3].

The knowledge that cytokinins play a key role in the regulation of plant growth and development has led to the assumption that they, through a common signal transduction system, may also have potential utility for the treatment of human diseases. Also, it has been proposed that the activities of cytokinins are associated with their capacity to lower the oxidative stress by interacting with biological regulators of oxidative stress [2,4]. We have recently made some contributions to this research area by investigating the radical scavenging activities of four natural N^6^-adenines (CKs) and the corresponding N^9^-ribosides (CKRs) [5,6] against synthetic and biologically relevant radicals.

Many studies have been conducted to examine the biological activities of natural cytokinins, and in addition to numerous prospective therapeutic applications, many authors have reported a wide range of pharmacologic and health-promoting properties, including neuroprotective, immunomodulatory, and anti-proliferative effects [7]. Although there are few studies that highlight the endogenous detection of cytokinins, there are numerous reports outlining the therapeutic potential of cytokinins in exogenous applications [8]. However, the effective concentrations of exogenously applied cytokinins that invoke various cellular responses are not well standardized since the understanding of the interaction between cytokinins and human cellular pathway targets is limited. So far, cytokinin nucleosides, in particular N^9^-ribosides (CKRs), have been characterized to have pleiotropic biological effects, including anti-tumor and anti-angiogenic activities, both in vitro and in vivo and have become potential candidates for treating a variety of cancers [9,10], representing valuable preclinical models to identify potential therapy targets and pharmacologically useful compounds. Thus, much emphasis has been placed on assessing new CKs with cytotoxic effects on cancer cell lines. In a comprehensive study of the cytotoxic effects of almost 50 natural cytokinins on human cell lines derived from diverse malignancies, Strnad’s group confirmed the high cytotoxic activities of CKRs with respect to their corresponding nucleobases [11]. Whether treatments resulted in cell cycle blockade and/or apoptosis, the result was strongly dependent on the cell line and the cytokinin used. Another general observation was related to the activity of CKRs, which were reported to be effective at submicromolar concentrations against various hematological malignant cell lines or at micromolar concentrations against other hematological malignant cells and cells derived from solid tumors [10]. Unfortunately, unlike the results from human neoplastic cell lines, studies reporting the cytotoxic activities of cytokinins toward normal human diploid cells are few and, like those designed to assess cytotoxicity toward neoplastic cell lines, are largely dependent on the cell type, the applied concentration, and type of cytokinin used. According to the published results, the most frequently used human cell culture toxicity assays in cytokinin screening are cell viability assays, providing a quantitative estimation of the viable cells in a culture [12,13,14]. On the other hand, the tests giving insights into direct cytotoxic effects and providing data on the safety and efficacy of CKs and CKRs in normal human cells metabolism are still insufficient [15,16,17]. For example, in vitro studies showed that K exhibited a biphasic effect at higher and lower concentrations; K mediates protective effects at low concentrations (below 100 nM), reducing apoptosis and protecting cells from oxidative stress-mediated cell death, but promotes cytotoxicity and genotoxicity in various cell types at higher concentrations (500 nM and above) [4,13]. Apart from in vitro assessments of cytotoxic effects, multiple in vitro and in vivo studies evaluated the applicability of cytokinin bases in skin protection (for example, K is the principal ingredients of several marketed cosmeceuticals), exploring various parameters relevant for aging amelioration and/or age-related disease therapy ([9,18,19] and the references cited therein). Few cytokine bases (K, *p*-T and iPA) have shown cytoprotective and anti-aging activities with optimal effects at 80 μM, which were evaluated further by assessing the safety and efficacy profiles of their topical application at a concentration of 0.1% in multiple open-label, single-arm clinical trials [9,19].

The literature data also indicate the roles of exogenously applied cytokinins in altering the level of host resistance to pathogens [20,21,22,23,24,25,26]. Recent investigations suggest that cytokinins play important roles in plant immunity [27,28,29,30,31,32] and are involved in the regulatory effects of defense responses on the direct growth, development, and virulence of non-cytokinin-producing phytopathogens [33,34,35] by suppressing bacterially induced hypersensitive response symptoms and by increasing antioxidant enzyme levels [33,36]. The elucidation of cytokinin-mediated cellular responses in microbial metabolism are still being researched, unquestionably representing an area in which any contribution is of certain value as it can greatly encourage the development of alternative strategies in the treatment of microbial infections. This issue is of particular importance since the rise in antimicrobial resistance poses a significant threat worldwide, diminishing the efficacy of common antimicrobial drugs against widespread infections. It is estimated that by 2050, antimicrobial resistance may become the world’s leading cause of death without preventative measures; this “silent pandemic” requires urgent attention and a coordinated approach across the human health, animal and environmental sectors, and food production. In order to improve knowledge for designing new tools and solutions for the effective prevention, detection, and treatment of drug-resistant infections, the development of new antimicrobial agents around proven natural scaffolds is considered the best short-term solution to the rising crisis of antimicrobial resistance [37].

Considering the evidence from numerous reports outlining the therapeutic potential of cytokinins in exogenous applications, it is surprising how only a few studies have evaluated their antimicrobial activities. Fleysher et al. [38,39,40] have evaluated the biological activities of aliphatic, aromatic, and alicyclic adenines and adenosines in vitro against cultures of *Streptococcus faecalis* and *Escherichia coli*, evidencing distinct responses. While most of aromatic adenosine analogs inhibited the growth of *E. coli* (IC_50_ = 10^−6^ to 10^−7^ M), among N^6^-aliphatic derivatives only adenosines with an N^6^-substituted *trans*- double bond demonstrated a greater *E. coli* inhibitory effect (IC_50_ = 10^−4^–10^−6^ M). In *S. faecalis* however, the aromatic-substituted adenosines showed marked stimulation of growth while the growth of the microorganism was affected only by the N^6^-propyl- and N^6^-allyladenosines (IC_50_ = 5 × 10^−4^ M and IC_50_ = 8 × 10^−5^ M, respectively). Degani et al. [41] have demonstrated the considerable suppression of *Harpophora maydis* fungal colony growth in vitro by kinetin (K). The addition of an exogenous cytokinin (K) to the culture media stimulated the growth of *Bacillus megaterium*, *E. coli*, *Staphylococcus aureus,* and *Erwinia carotovora*; stimulated conidial germination in *Erysiphe graminis* [42,43,44,45]; inhibited the growth of *Corynebacterium michiganense* [43] and germ tube growth in *Erysiphe graminis* [45]; and had little effect on growth of *Saprolegnia australis* Elliott [46], *Saccharomyces cerevisiae* Meyen, and *Schizosaccharomyces pombe* [42].

**Figure 1:**
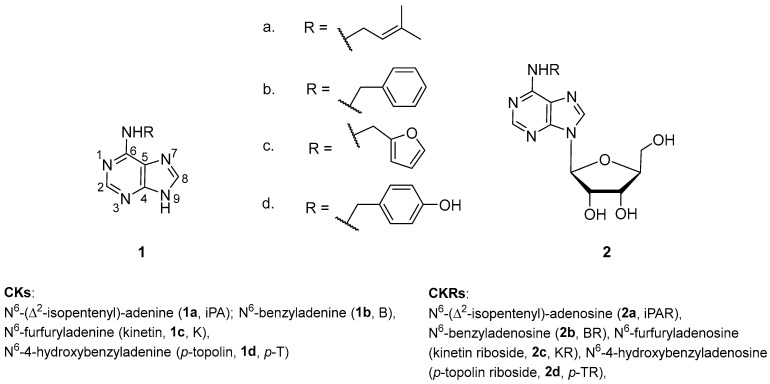
Chemical structures of cytokinins: free bases (CKs—iPA, B, K and *p*-T) and ribosides (CKRs—iPAR, BR, KR and *p*-TR) examined in this paper.

Considering previous reports on the antimicrobial potential of cytokinins, we designed this study to evaluate the in vitro antimicrobial activities of selected natural cytokinins (Figure 1), both CKs and CKRs, against common human pathogens and explore their binding interactions with key fungal enzymes using molecular docking, with the aim of identifying promising candidates for the development of new antifungal agents.

## 2. Results and Discussion

The antimicrobial activities of four naturally occurring cytokinin bases (CKs—N^6^-(Δ^2^-isopentenyl)adenine (iPA), N^6^-benzyladenine (B), N^6^-furfuryladenine (kinetin, K), and N^6^-4-hydroxybenzyladenine (*p*-topolin, *p*-T)) and their corresponding N^9^-ribosides (CKRs—iPAR, BR, KR, and *p*-TR) were evaluated by determining well-known endpoints of microbial susceptibility, the minimum inhibitory concentration (MIC) and the minimum microbicidal concentration, which include the minimum bactericidal concentration (MBC) and minimum fungicidal concentration (MFC). Microbial susceptibility testing was performed using the broth microdilution method [47]. Activities of the selected natural CKs and CKRs were evaluated against seven bacteria (*Escherichia coli* ATCC 25922, *Proteus vulgaris* bacterial isolate, *Salmonella enterica* ATCC 13076, *Bacillus subtilis* ssp. *spizizenii* ATCC 6633, *Enterococcus faecalis* ATCC 29212, *Listeria monocytogenes* ATCC 13932, and *Staphylococcus aureus* ATCC 25923) and two fungal strains (mold species *Aspergilus brasiliensis* ATCC 16404 and yeast species *Candida albicans* ATCC 10231). The results obtained from the broth microdilution assay are presented in Table 1.

It would be important to mention that, according to the authors’ best knowledge and based on a literature search [48], none of previously reported studies included the *A. brasiliensis*/*C. albicans* strains or most of the human pathogens assayed here in the bioassay evaluating the antimicrobial activities of the selected natural CKs and CKRs.

The selected CKs and CKRs showed a wide range of activities, depending on the strain tested. Two Gram-positive microorganisms (*L. monocytogenes* and *S. aureus*) and all selected Gram-negative microorganisms (*E. coli*, *P. vulgaris,* and *S. enterica*) were completely resistant to the CKs and CKRs at the tested concentrations. All of the compounds interfered with the growth of Gram-positive *B. subtilis* ssp. *spizizenii* and *E. faecalis*; for both bacterial strains, the N^9^-ribosides were slightly more inhibitory than the corresponding free bases (Table 1). The enhanced activity of ribosides against Gram-positive bacteria is entirely consistent with known biological mechanisms: Gram-positive bacteria possess a thick but porous peptidoglycan layer that facilitates the diffusion of larger molecules, including ribosides. In contrast to the results observed for *B. subtilis* ssp. *spizizenii* and *E. faecalis*, for the two tested fungal strains, *A. brasiliensis* and *C. albicans*, the free cytokinin bases were slightly more inhibitory than the corresponding N^9^-ribosides. Also, more pronounced differential responses within the microbial systems of fungal strains to the aromatic N^6^-substituted adenosines and its N^9^-ribosides could be observed, ranging from completely inactive, as in case of *p*-T and *p*-TR, to highly susceptible, as could be observed for K and KR.

It is interesting to note that in our experiments, *p*-T and *p*-TR did not affect the growth of most of the assayed microorganisms. However, like the other cytokinins tested, they inhibited the growth of *B. subtilis* and *E. faecalis* at the limits of the tested concentrations (MIC = 3.9 μg/mL), reaching an MIC value almost comparable to the positive control we used (Table 1, reference antibiotic MIC = MBC = 1.56 μg/mL and MIC = MBC = 6.25 μg/mL, respectively). A surprising result was that all CKs/CKRs selected for this study showed comparable *B. subtilis* and *E. faecalis* antimicrobial effects, acting as bacteriostatic agents.

Regarding the antifungal activity, CKs and CKRs were more effective as anticandidal agents (Table 1), while the activity toward *A. brasiliensis*, if present, was much more modest. The best results derived from the current antimicrobial assay were the results obtained for K on *C. albicans*, which was characterized by superior performance compared to all other tested samples and with an MIC comparable to the antimycotic nystatin that was used as the reference standard (Table 1, MIC = 3.9 μg/mL and MIC = 6.25 μg/mL, respectively). Of at least the same importance are the results obtained for the corresponding nucleoside KR toward *C. albicans*, whose inhibitory concentration was twice as high, but with an MIC still comparable to the results obtained for the antimycotic nystatin (Table 1, MIC = 7.8 μg/mL and MIC = 6.25 μg/mL, respectively). Another compound exerting good fungistatic potential was B, followed by its N^9^-riboside, BR, and both were again more potent against *C. albicans* (Table 1, MIC = 62.5 μg/mL and MIC = 125 μg/mL, respectively). Unlike the cytokinins listed above, iPA and iPAR were effective against *C. albicans* (MIC = 125 μg/mL and MFC = 500 μg/mL, respectively), but had no effect on the growth of the other tested fungal organism—the mold *A. brasiliensis* (Table 1).

The results from our experiment reveal that CKs and CKRs inhibit the growth of *C. albicans* and *A. brasiliensis* in a dose-dependent manner. Due to the minor effect CKs and CKRs had on *A. brasiliensis*, we focused our attention on *C. albicans*.

Although few studies have discussed effects of cytokinins on the growth of phytopathogenic fungi, no particular mechanisms have been reported [49], except that Gupta et al. [34] described increased CK levels inhibiting the growth, development, and virulence of *Botrytis cinerea*, *Sclerotium rolfsii*, and *Fusarium oxysporum* f. sp. *lycopersici* in tomato plants by targeting the cell cycle and downregulating the cytoskeleton or endocytic trafficking. At present, the molecular targets of the studied CKs/CKRs and the explanation for their observed significant anticandidal activities remain elusive. Using computational studies, we attempted to elucidate the “binding” modes that could aid future development toward more effective anticandidal “leads”.

Fungal infections, or mycosis, are diseases caused by a fungus (yeast or mold) and can cause invasive diseases that can even lead to death. Among the most drug-resistant fungal forms are those belonging to the *Candida* and *Aspergillus* spp.

In recent years, epidemiological data have recorded an upsurge of fungal infections that are increasingly resistant to antimycotic drug treatments [50].

To counteract the phenomenon of rapidly evolving drug resistance, alternative strategies in the treatment of fungal infection are necessary, which often involve synthetic approaches to develop new antimicrobial agents based on the privileged scaffolds of azoles, which so far are the only class of antifungals approved for standalone systemic use [51]. The reliance on natural products to provide novel molecular entities is well established [52], and it is not surprising that a large proportion of the currently applied antimicrobial agents are derived from natural products. Treatment options for fungal infections in humans are currently limited to five different classes of antifungal drugs, only three of which are regularly used as standalone treatments for mycosis [50]. In addition to the treatment of mycosis with small-molecule drugs, the so-called non-traditional antifungals are also being evaluated by researchers. So far, peptides, antibodies, vaccines, immunomodulatory compounds, mycophages, and virulence factor inhibitors have been included into the concept of “non-traditional antifungals” [51]. Among the important virulence factors that enable *C. albicans* to invade the host tissues and evade host defense mechanisms are hydrolytic enzymes, most of which are extracellularly secreted by fungi. Aspartic proteinases (SAPs) are the most significant and the best-studied representatives of extracellular hydrolytic enzymes secreted by *C. albicans* to degrade host proteins by digesting or destroying cell membranes and by degrading host surface molecules. The role of SAPs has been intensively investigated during the last decades and these enzymes are considered essential in the development of an effective pharmacological treatment specifically modulated to prevent and control *Candida* infections [53]. Folate biosynthesis has been effectively targeted to develop antibacterial, antiprotozoal, and antineoplastic therapies, and a few promising attempts directed toward fungal-specific inhibitors targeting this pathway have been made so far [54]. Dihydrofolate reductase (DHFR) catalyzes the conversion of dihydrofolate to tetrahydrofolate and is an essential cofactor for DNA synthesis and a well-characterized drug target. DHFR inhibitors are already used to treat a wide variety of diseases, including as antimalarial (trimethoprim), anticancer (methotrexate), and antifungal treatments, such as pyrimethamine in the treatment of *Pneumocystis jirovecci* pneumonia [55]. Recently, DHFR was validated as a target for the development of antimicrobials against several *Candida* species, including *C. albicans* and *C. glabrata*, with the results confirming the validity of targeting DHFR and other enzymes in the folate biosynthetic pathway as a strategy for designing new and effective therapies to combat invasive fungal infections [56]. Therefore, we applied in silico molecular docking studies to determine the interactions and binding energies between CKs and CKRs and the most prominent virulence factors/antimicrobial enzymes in *Candida* spp. (*C. albicans*).

In Table 2, numerical values for all calculated scoring functions are presented. Based on the MolDock score and Reranked score, the molecule K demonstrated the highest binding potential with amino acids in the active sites of both studied targets. Conversely, the molecule *p*-T showed the lowest binding potential according to both scoring metrics. The results for other scoring functions mirrored this pattern. For instance, VdW scores for the molecule K were −44.2689 kcal/mol and −48.9838 kcal/mol for SAPs and DHFR, respectively, while for the molecule *p*-T, these values were −25.0538 kcal/mol and −22.4494 kcal/mol. These results indicate that the molecule K exhibits stronger van der Waals interactions, contributing to a higher binding potential. This pattern holds for the other scoring functions as well.

The strong in vitro anticandidal activities of K and KR are consistent with their superior binding affinities for SAP3 and DHFR, suggesting that these interactions may underlie their observed biological effects. The molecular docking study results identified hydrogen bonds, hydrophobic interactions, and hydrophilic interactions between the studied molecules (CKs and CKRs) and amino acids within the binding pockets of both SAPs and DHFR. The preferred geometric orientation (molecular pose) within the enzyme active site is shown in Figure 2, with two-dimensional representations available in the Supplementary Information (Figures S1–S16).

The most active compounds, K and KR, demonstrated the highest predicted binding affinities, forming multiple stabilizing interactions such as hydrogen bonding, π–π stacking, and hydrophobic contacts. These interactions likely contribute to their enhanced binding stability within the active sites of SAP3 and DHFR. In contrast, compounds with lower antifungal activity exhibited fewer such interactions and weaker docking scores, supporting the idea that target affinity may underlie at least part of the observed antifungal activity.

The results for SAP indicate that the molecule K adopted a unique binding position compared to other molecules, aligning well with the experimental results, as varying binding positions can affect kinetics. Molecule K formed conventional hydrogen bonds with Tyr128 and Leu194, while Asp32 and Asp86 formed hydrogen bonds with all other molecules. Additionally, Tyr84 showed π–π stacking interactions with molecules B, iPA, KR, and *p*-T and δ–π interactions with iPAR and *p*-TR.

For DHFR, molecules with an added sugar group showed unfavorable acceptor–acceptor interactions with Tyr118, but also formed conventional hydrogen bonds with Ile9, Ile19, and Ala11. Lys57 exhibited δ–π interactions and Ile117 showed π–alkyl interactions with all studied molecules. The analysis of these results indicates that K and KR are capable of binding to the active sites of the tested enzymes and form favorable interactions with key amino acids.

The in vitro results, supported by the molecular docking studies, suggest that the natural cytokinins K and KR could represent the starting framework for the design of new molecules endowed with the potential for the development of naturally based antimycotics.

It is important to note that our findings are based on in vitro assays and in silico modeling. Therefore, further studies are warranted to validate the observed effects in vivo, assess the pharmacokinetic and toxicological profiles of these compounds, and elucidate the molecular mechanisms underlying their antifungal actions. Despite these limitations, the promising activities of K and KR suggest their potential as starting points for the development of novel antifungal agents.

As with many in vitro studies, a limitation of our work is the absence of in vivo validation. While the antimicrobial effects of cytokinins, especially K and KR, are promising, further in vivo experiments are necessary to confirm their efficacy, assess their pharmacokinetics and toxicity, and evaluate their suitability as therapeutic agents. The translation of in vitro activity into clinical relevance requires detailed preclinical testing, which will be the focus of our future investigations. In addition to the promising antifungal activity observed against planktonic *C. albicans*, future studies will also focus on evaluating the effects of selected cytokinins on biofilm formation and mature biofilm integrity, which represent major virulence mechanisms and therapeutic challenges in clinical settings.

## 3. Materials and Methods

### 3.1. Reagents and Materials

The following CKs, N^6^-(Δ^2^-isopentenyl) adenine (iPA), N^6^-benzyladenine (B), kinetin (K), and p-topolin (*p*-T), and their correspondent ribosides, N^6^-(Δ^2^-isopentenyl)adenosine (iPAR), N^6^-benzyladenosine (BR), kinetin riboside (KR), and *p*-topolin riboside (*p*-TR), were obtained from OlChemIm Ltd., (Olomouc, Czech Republic), and were of analytical grade. Unless specified otherwise, all reagents and standards were purchased from Merck (Darmstadt, Germany).

### 3.2. Microbial Strains

The testing of the natural cytokinins’ efficacy was performed against eight American Type Culture Collection (ATCC) reference strains and one isolate. The panel of microorganisms applied for the purpose of this study included four Gram-positive bacteria (*Bacillus subtilis* ssp. *spizizenii* ATCC 6633, *Enterococcus faecalis* ATCC 29212, *Listeria monocytogenes* ATCC 13932, and *Staphylococcus aureus* ATCC 25923), three Gram-negative bacteria (*Escherichia coli* ATCC 25922, *Salmonella enterica* ATCC 13076, and *Proteus vulgaris* (isolate from food), and two fungal organisms: one yeast strain (*Candida albicans* ATCC 10231) and one mold (*Aspergilus brasiliensis* ATCC 16404) strain. The *P. vulgaris* food isolate was obtained from the Veterinary Specialistic Institute Niš, Niš, Serbia, and is stored in the in-house microbiological collection. All microorganisms were maintained at −20 °C under appropriate conditions and regenerated twice before further use in the manipulations.

### 3.3. Screening of Antimicrobial Activities by the Microdilution Method

The antimicrobial activity was evaluated using a broth microdilution assay [47]. The minimum inhibitory concentration (MIC) determinations were performed by a serial dilution method in 96-well microtiter plates using the method of Sarker et al. [57], with slight modifications [58]. As the MIC and MBC/MFC are discrete endpoint values, all were obtained as constants across experimental replicates, and no statistical processing (e.g., ANOVA) was performed. This is in line with the standard protocol of broth microdilution assays in which MICs and MFCs are used as direct qualitative and semi-quantitative indicators of antimicrobial potency. Due to the nature of the broth microdilution assay, antimicrobial activity was determined based on discrete endpoints (MICs and MBCs/MFCs). This method does not generate continuous data suitable for plotting dose–response curves. Overnight cultures of the tested strains were suspended in sterile physiological saline. Mueller–Hinton broth (Thermo Scientific^TM^, Lenexa, KS 66215, USA) and Yeast Broth (Thermo Scientific^TM^, Lenexa, KS 66215, USA) were inoculated with bacterial/yeast suspensions, and their turbidity was adjusted to 0.5 on the McFarland scale using a DEN-1 McFarland densitometer (Biosan Riga, Latvia). The final density of bacterial and yeast inocula corresponded to 5 × 10^5^ CFUs (colony forming units). A suspension of the mold (*A. brasiliensis*) was made in Sabouraud dextrose broth (SDB) and its turbidity was confirmed by viable counting in a Thoma chamber with 2 × 10^5^ CFUs as the final size of the inoculum. Dimethyl sulfoxide (100 mg/mL, aqueous solution) was used to dissolve and dilute the cytokinin samples to the concentration of the stock solutions (1000 µg/mL). The inocula were added to all wells of a 96-well plate, in which serial dilutions of the samples was prepared. The final concentrations of tested samples adopted to evaluate antibacterial/antifungal activities ranged from 1.00 to 500 µg/mL. Three columns in each plate were used as controls. Two were used as positive controls, containing a broad-spectrum antibiotic (doxycycline in a serial dilution of 0.02–50 μg/mL) to determine the sensitivity of Gram-negative and Gram-positive bacterial species and an antimycotic (nystatin in a serial dilution of 0.02–50 μg/mL) to determine the sensitivity of fungal/mold species. The third column contained the solvent as a negative control. Microbial growth was determined by adding 10 µL of a resazurin (BHD Laboratory Supplies, Poole, UK) indicator solution (prepared by dissolving a 270 mg tablet in 40 mL of sterile distilled water) to each microtiter plate well. The plates were prepared in triplicate and incubated at 37 °C for 24 h (bacteria) or at 30 °C for 48 h (fungi/mold). The color change was then assessed visually. Any color changes from purple to pink or colorless after the addition of resazurin were recorded as positive. The lowest concentration at which the color change occurred was taken as the MIC value. Tests were carried out in triplicate.

The minimal bactericidal/fungicidal concentration (MBC/MFC) was determined by spreading the content of each microtiter plate well (50 µL) in which a color change occurred on sterile nutrient agar NA (bacteria) and SDA (fungi/mold) set in Petri dishes [47]. These plates, after standing at 4 °C for 2 h to allow dispersal, were incubated at 37 °C for 24 h for bacteria or at 30 °C for 48 h for fungi. The MBC/MFC was taken as the lowest concentration of the tested substance at which 99.9% of inoculated microorganisms were killed. Tests were carried out in triplicate.

The average of three values of three repetitions was calculated, and was presented as the MICs or MBCs/MFCs for the samples tested and bacterial strains (Table 1).

### 3.4. Molecular Docking

Molecular docking simulations were performed using Molegro Virtual Docker (MVD v. 2013.6.0.1), which applies the MolDock scoring function based on a piecewise linear potential (PLP), allowing an efficient approximation of steric and hydrogen bonding energies with high predictive accuracy. The ligands were first drawn and geometrically optimized using the MMFF94 force field within Sybyl-X 2.1.1, which was also used to assign partial atomic charges. Protonation states were adjusted to reflect physiological pH (~7.4). The choice of targets was guided by their established roles in *C. albicans* pathogenesis. Secreted aspartic proteinase 3 (SAP3) belongs to a family of enzymes that contribute to tissue invasion, the degradation of host proteins, and immune evasion, thereby acting as key virulence factors. Dihydrofolate reductase (DHFR) is an essential enzyme involved in folate metabolism and nucleotide biosynthesis, making it a critical target for fungal survival and proliferation. Both proteins have been validated as antifungal drug targets in previous studies [59,60], and were thus selected to evaluate potential interactions with active cytokinins. The crystal structure of SAP3 (secreted aspartic proteinase 3) was obtained from the Protein Data Bank (PDB ID: 2H6T), while the structure of *C. albicans* dihydrofolate reductase (DHFR) was based on a validated homology model previously reported in the literature. Prior to docking, all protein structures were processed by removing crystallographic water molecules and co-crystallized ligands, and missing residues were rebuilt using the built-in tools in MVD. Binding sites were defined as spherical regions with a 15 Å radius centered around the native ligand or catalytic core residues. A rigid receptor/flexible ligand approach was applied. The docking search algorithm employed a maximum of 1500 iterations, with a population size of 50, crossover rate of 0.9, scaling factor of 0.5, energy threshold of 100, and 10 docking runs per compound. To validate the docking protocol, re-docking of co-crystallized ligands was performed, yielding RMSD values below 2.0 Å, confirming the reliability of the approach. Docked complexes were analyzed both energetically and visually. Primary scoring was based on the MolDock score [61], which integrates intermolecular interaction energies, while the Reranked score accounted for both intermolecular and intramolecular components in a refined post-docking evaluation. In addition, van der Waals, hydrogen bonding, and steric interaction energies were calculated and reported to aid in the qualitative interpretation of the binding strength. The best-scoring poses were selected for each ligand and inspected for key interactions within the binding sites. The two-dimensional interaction diagrams were generated using Discovery Studio Visualizer v20.1.0.19 and are provided in the Supplementary Information (Figures S1–S16). The numerical docking results are summarized in Table 2 of the main text.

## 4. Conclusions

This study demonstrated that certain natural cytokinins, particularly kinetin (K) and its riboside (KR), possess significant in vitro antifungal activity against *Candida albicans*, with MIC values comparable to the reference antifungal drug nystatin. The tested cytokinins exhibited modest or no activity against Gram-negative bacteria, while a consistent bacteriostatic effect was observed against *Bacillus subtilis* ssp. *spizizenii* and *Enterococcus faecalis*. Molecular docking studies revealed that K and KR form favorable interactions with two validated antifungal targets in *C. albicans*, secreted aspartic proteinase (SAP3) and dihydrofolate reductase (DHFR), which may contribute to their observed antifungal effects. Taken together, the data suggest that K and KR may serve as starting points for the design of cytokinin-based antifungal agents. Further studies are needed to assess their bioavailability, toxicity, and in vivo efficacy.

## Figures and Tables

**Figure 2:**
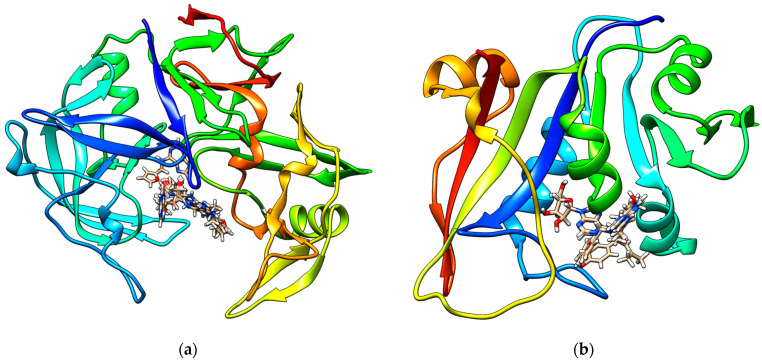
Best calculated poses for all studied molecules within the active sites of (**a**) secreted aspartic proteinase (SAP) 3 and (**b**) dihydrofolate reductase (DHFR).

**Table 1:** Minimal inhibitory concentrations (MICs) and minimal bactericidal/fungicidal concentrations (MBCs/MFCs) of the selected CKs (iPA, B, K, and *p*-T) and CKRs (iPAR, BR, KR, and *p*-TR). NT—not tested, /—not sensitive in the range of the tested concentrations.

Tested Compound	Bacterial Strains				Fungal Strains					
	*B. subtilis* ssp. *spizizenii*		*E. faecalis*		*A. brasiliensis*		*C. albicans*			
	MIC ^1^		MIC		MIC = MFC ^2^		MIC		MFC	
	μg/mL	nM	μg/mL	nM	μg/mL	nM	μg/mL	nM	μg/mL	nM
iPA	3.9	19.2	3.9	19.2	/	/	125	615	500	2460
iPAR	3.9	11.6	3.9	11.6	/	/	125	327.7	500	1490.9
B	3.9	17.3	3.9	17.3	500	2219.8	62.5	277.5	500	2219.8
BR	3.9	10.9	3.9	10.9	/	/	125	349.8	500	1399.1
K	3.9	18.1	3.9	18.1	250	1161.6	3.9	18.1	250	1161.6
KR	3.9	11.2	3.9	11.2	250	719.8	7.8	22.5	500	1439.6
*p*-T	3.9	16.2	3.9	16.2	/	/	/	/	/	/
*p*-TR	3.9	10.4	3.9	10.4	/	/	/	/	/	/
Positive control	MIC = MBC ^3^		MIC = MBC		MIC = MFC		MIC = MFC			
	μg/mL	nM	μg/mL	nM	μg/mL	nM	μg/mL		nM	
Doxycycline ^4^	1.56	3.5	6.25	14.1	NT	NT	NT		NT	
Nystatin ^5^	NT	NT	NT	NT	0.78	0.8	6.25		6.7	

^1^ The minimal inhibitory concentration (MIC) is the lowest concentration of an antimicrobial agent required to prevent visible growth of a specific microorganism, ^2^ the minimum fungicidal concentration (MFC) is the minimum concentration of an antimicrobial capable of inactivating (killing) more than 99.9% of the inoculated fungi, and ^3^ the minimum bactericidal concentration (MBC) is deemed to be the minimum concentration of an antimicrobial capable of inactivating (killing) more than 99.9% of the inoculated bacteria; ^4^ reference antibiotic and ^5^ reference antimycotic.

**Table 2:** Scores (kcal/mol) for all studied compounds designed with the aid of a computer.

	Molecule	Steric Energy ^1^	VdW ^2^	H-Bond ^3^	No H-Bond 90 ^4^	Energy ^5^	MolDock Score ^6^	Reranked Score ^7^
Aspartic proteinase	iPA	−93.9147	−29.1649	−14.5711	−19.9204	−100.884	−102.944	−80.9744
	iPAR	−99.1543	−33.5975	−7.0872	−9.10958	−102.893	−103.004	−86.5915
	B	−127.038	−42.2686	−10.2201	−13.5644	−134.303	−149.403	−111.304
	BR	−129.563	−37.6263	−10.4694	−16.405	−132.741	−147.791	−106.981
	K	−133.762	−44.2689	−12.0332	−13.9491	−144.948	−158.188	−119.256
	KR	−134.535	−43.4062	−12.4022	−18.9622	−142.191	−155.787	−115.098
	*p*-T	−90.7434	−25.0538	−3.23618	−5	−88.5194	−91.6294	−75.39
	*p*-TR	−98.7731	−32.0643	−6.8347	−7.97501	−94.1577	−96.2947	−81.197
Dihydrofolate reductase	iPA	−107.774	−31.1458	−7.14074	−9.47577	−105.264	−108.278	−87.6308
	iPAR	−99.8345	−30.0122	−6.7828	−8.91991	−106.092	−113.099	−86.9306
	B	−145.136	−43.0046	−18.3581	−19.7344	−154.594	−169.705	−127.109
	BR	−130.624	−41.7649	−17.9418	−19.9993	−148.073	−162.683	−121.438
	K	−141.037	−48.9838	−20.7666	−23.7065	−161.709	−182.815	−132.52
	KR	−150.046	−44.5959	−17.849	−19.8237	−159.49	−174.555	−132.708
	*p*-T	−93.0454	−22.4494	−11.211	−11.8636	−100.551	−102.045	−80.1823
	*p*-TR	−101.685	−30.1665	−9.19931	−15.0432	−105.502	−111.859	−85.6092

^1^ Steric energy parameter; ^2^ van der Waals energy parameter; ^3^ hydrogen bonding energy; ^4^ the energy from no hydrogen bond interactions; ^5^ binding energies of the molecules in the active sites of the enzymes. ^6^ the MolDock scoring function was set with a grid resolution of 0.30 A. It was set at a maximum iteration of 1500 with a simplex evolution size of 50 and a minimum of 10 runs were performed for each compound with threshold energy of 100. ^7^ The reranked score is a linear combination of E-inter (steric, van der Waals, hydrogen bonding, and electrostatic interactions) between the ligand and the protein, and E-intra (torsion, sp^2^–sp^2^, hydrogen bonding, van der Waals, and electrostatic interactions) of the ligand weighted by pre-defined coefficients.

## Data Availability

The data presented in this study are available upon request from the corresponding author.

## Supplementary Materials

The following supporting information can be downloaded at https://www.mdpi.com/article/10.3390/plants14121749/s1: Figure S1. Two-dimensional representation of the interactions between the molecule iPA and amino acids in the SAP active site; Figure S2. Two-dimensional representation of the interactions between the molecule iPAR and amino acids in the SAP active site; Figure S3. Two-dimensional representation of the interactions between the molecule B and amino acids in the SAP active site; Figure S4. Two-dimensional representation of the interactions between the molecule BR and amino acids in the SAP active site; Figure S5. Two-dimensional representation of the interactions between the molecule K and amino acids in the SAP active site; Figure S6. Two-dimensional representation of the interactions between the molecule KR and amino acids in the SAP active site; Figure S7. Two-dimensional representation of the interactions between the molecule *p*-T and amino acids in the SAP active site; Figure S8. Two-dimensional representation of the interactions between the molecule *p*-TR and amino acids in the SAP active site; Figure S9. Two-dimensional representation of the interactions between the molecule iPA and amino acids in the DHFR active site; Figure S10. Two-dimensional representation of the interactions between the molecule iPAR and amino acids in the DHFR active site; Figure S11. Two-dimensional representation of the interactions between the molecule B and amino acids in the DHFR active site; Figure S12. Two-dimensional representation of the interactions between the molecule BR and amino acids in the DHFR active site; Figure S13. Two-dimensional representation of the interactions between the molecule K and amino acids in the DHFR active site; Figure S14. Two-dimensional representation of the interactions between the molecule KR and amino acids in the DHFR active site; Figure S15. Two-dimensional representation of the interactions between the molecule *p*-T and amino acids in the DHFR active site; and Figure S16. Two-dimensional representation of the interactions between the molecule *p*-TR and amino acids in the DHFR active site.
