# Supplementary material for: Exploring the Potential of Plant Cytokinins Against Common Human Pathogens: In Vitro Assessment and In Silico Insights
Source: Plants (Basel). 2025 Jun 7;14(12):1749. doi: 10.3390/plants14121749 (PMC12196393; doi:10.3390/plants14121749)
Supplement: Supplementary file 1 [file plants-14-01749-s001.zip › plants-3557373-supplementary.pdf]

Supplementary information

Exploring the potential of plant cytokinins against common human pathogens: in vitro assessment and in silico insights

Jelena Lazarević [1,*], Aleksandar Veselinović [1], Marija Stojiljković [2], Miloš Petrović[2], Pierangela Ciuffreda[3], and Enzo Santaniello[4]

[1] Department of Chemistry, Faculty of Medicine, University of Niš, Bulevar Dr Zorana Đinđića 81, 18000, Niš, Serbia; jelenalazarevic@medfak.ni.ac.rs, jelena217@yahoo.com, aveselinovic@medfak.ni.ac.rs
[2] Veterinary Specialistic Institute Niš, Dimitrija Tucovića 175, 18106 Niš, Serbia; milosvsinis@gmail.com
[3] Dipartimento di Scienze Biomediche e Cliniche "L. Sacco", Universita degli Studi di Milano, Milano, Italy; pierangela.ciuffreda@unimi.it
[4] Faculty of Medicine, University of Milano, Italy; enzo.santaniello@unimi.it, esantaniello44@gmail.com
* Correspondence: jelenalazarevic@medfak.ni.ac.rs, jelena217@yahoo.com; Tel.: +381-631045128

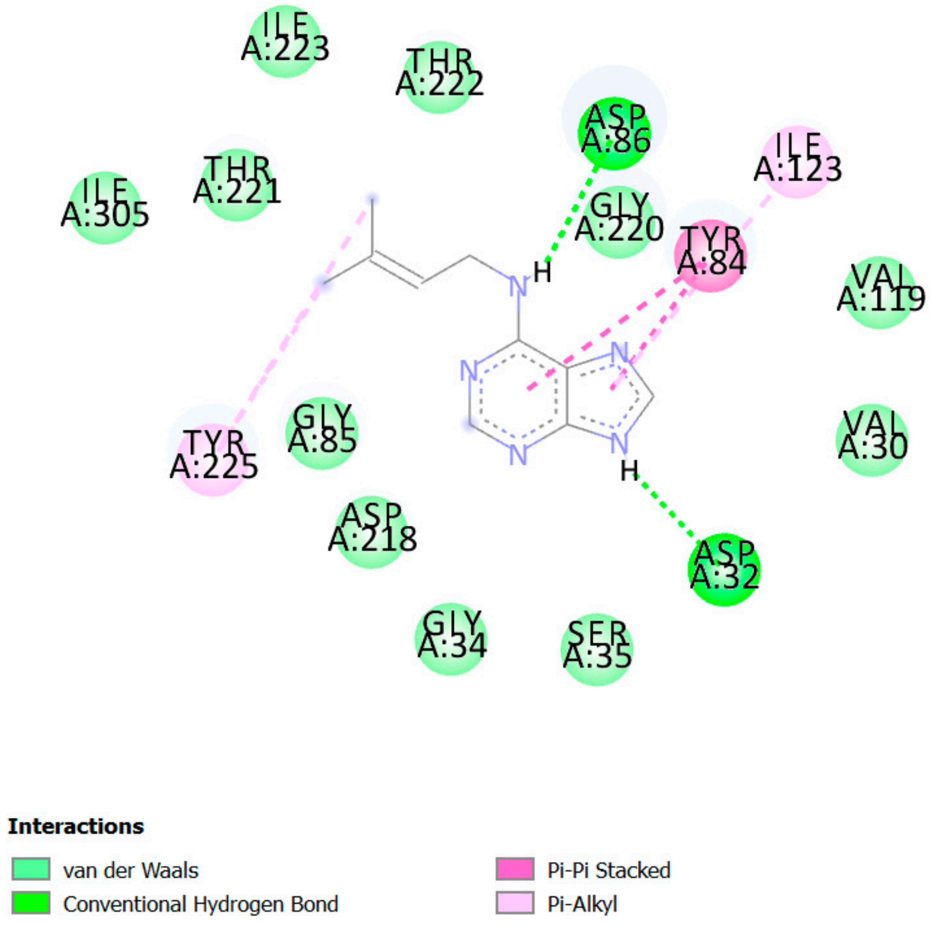

Figure S1. Two-dimensional representation of interactions between molecule **iPA** and amino acids from SAP active site.

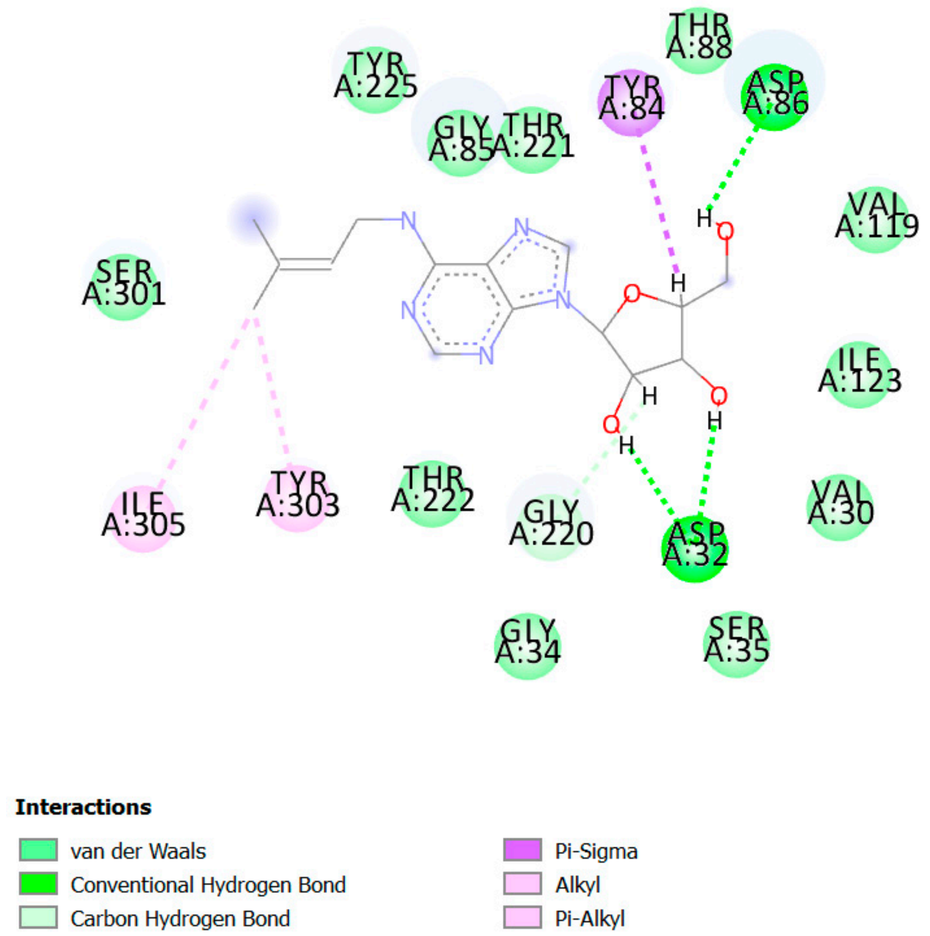

Figure S2. Two-dimensional representation of interactions between molecule **iPAR** and amino acids from SAP active site.

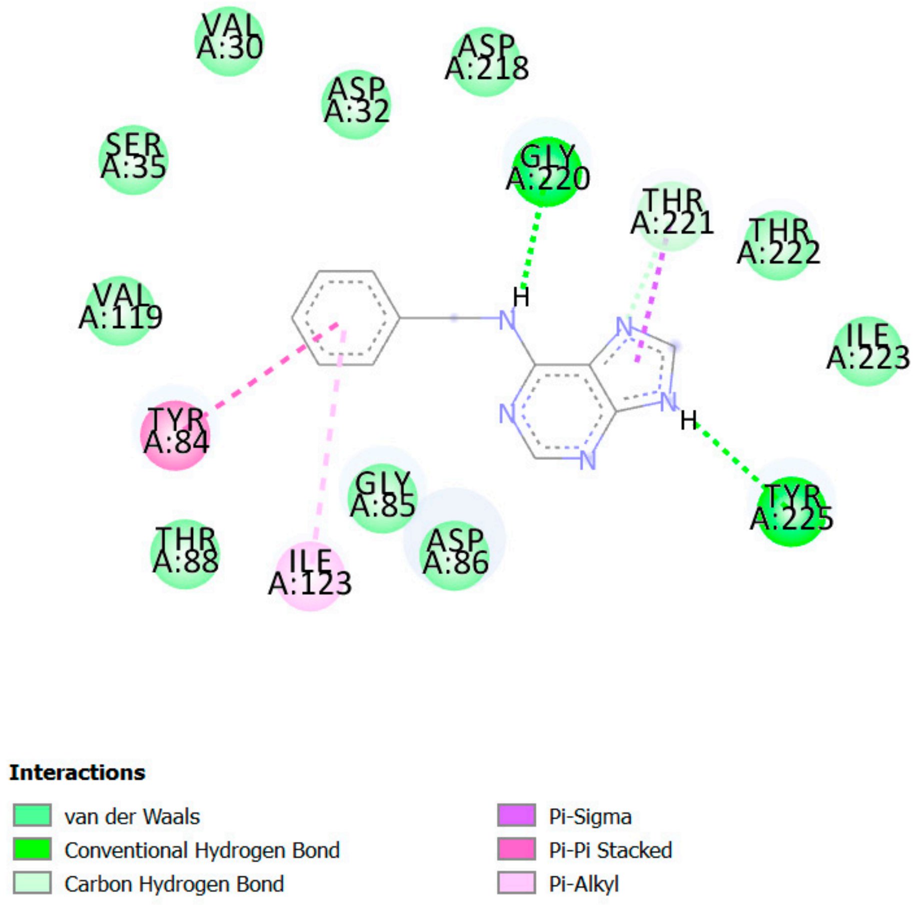

Figure S3. Two-dimensional representation of interactions between molecule **B** and amino acids from SAP active site.

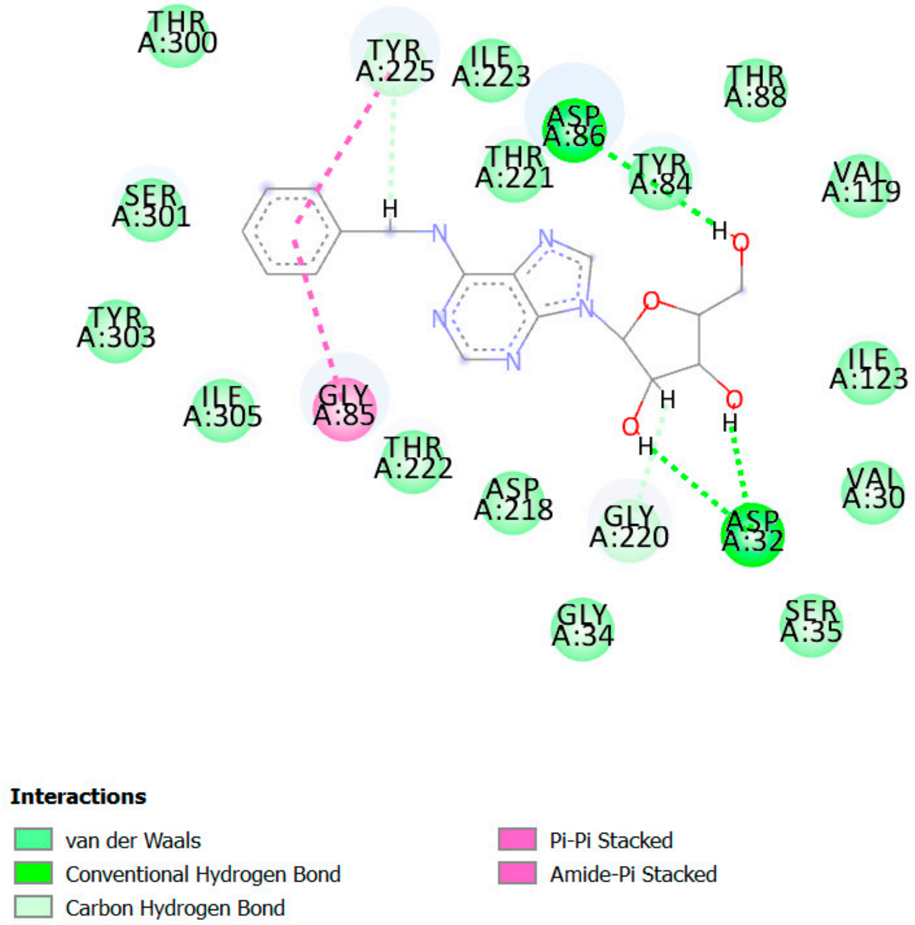

Figure S4. Two-dimensional representation of interactions between molecule **BR** and amino acids from SAP active site.

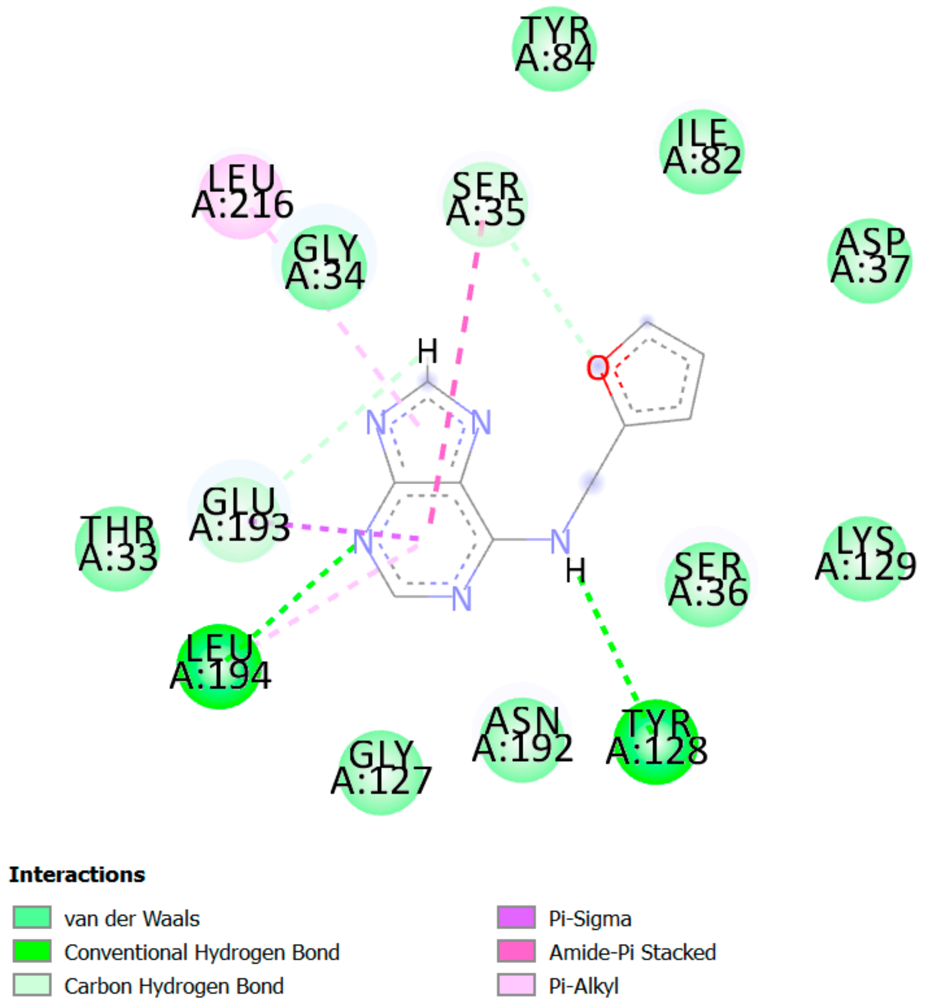

Figure S5. Two-dimensional representation of interactions between molecule **K** and amino acids from SAP active site.

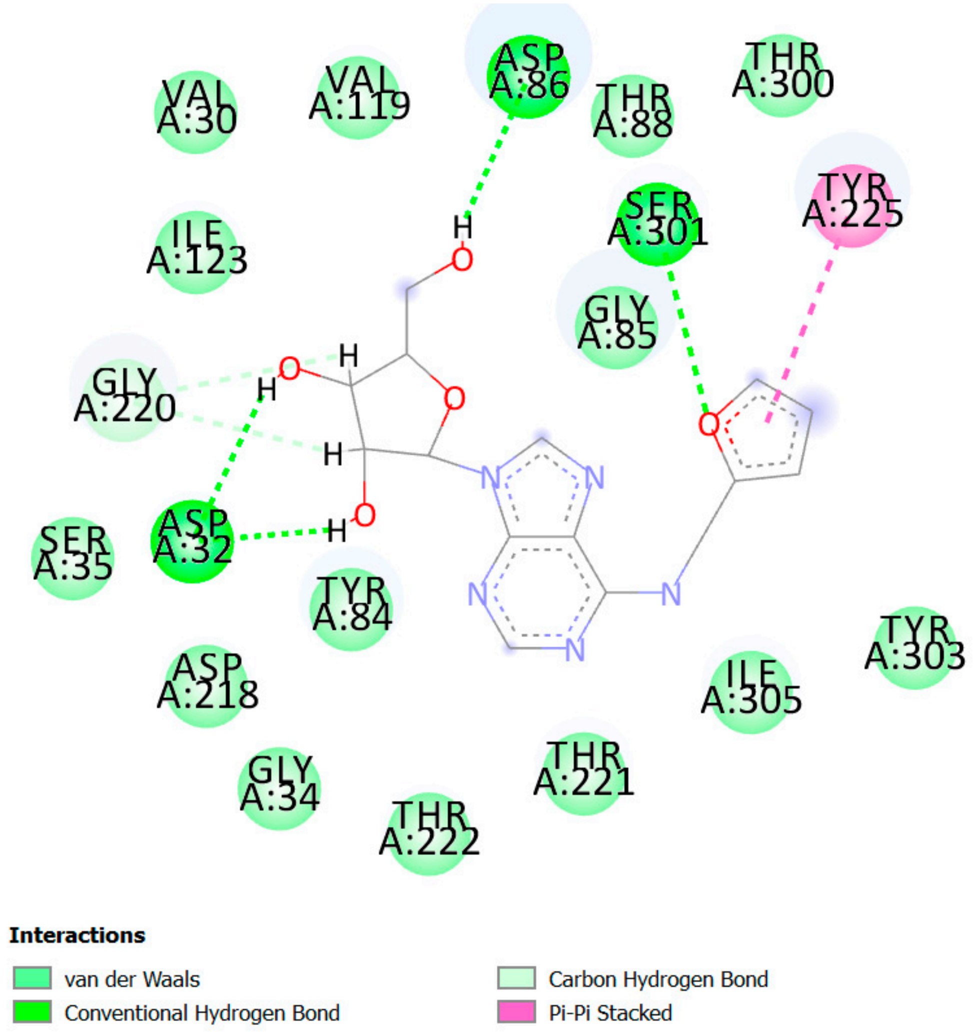

Figure S6. Two-dimensional representation of interactions between molecule **KR** and amino acids from SAP active site.

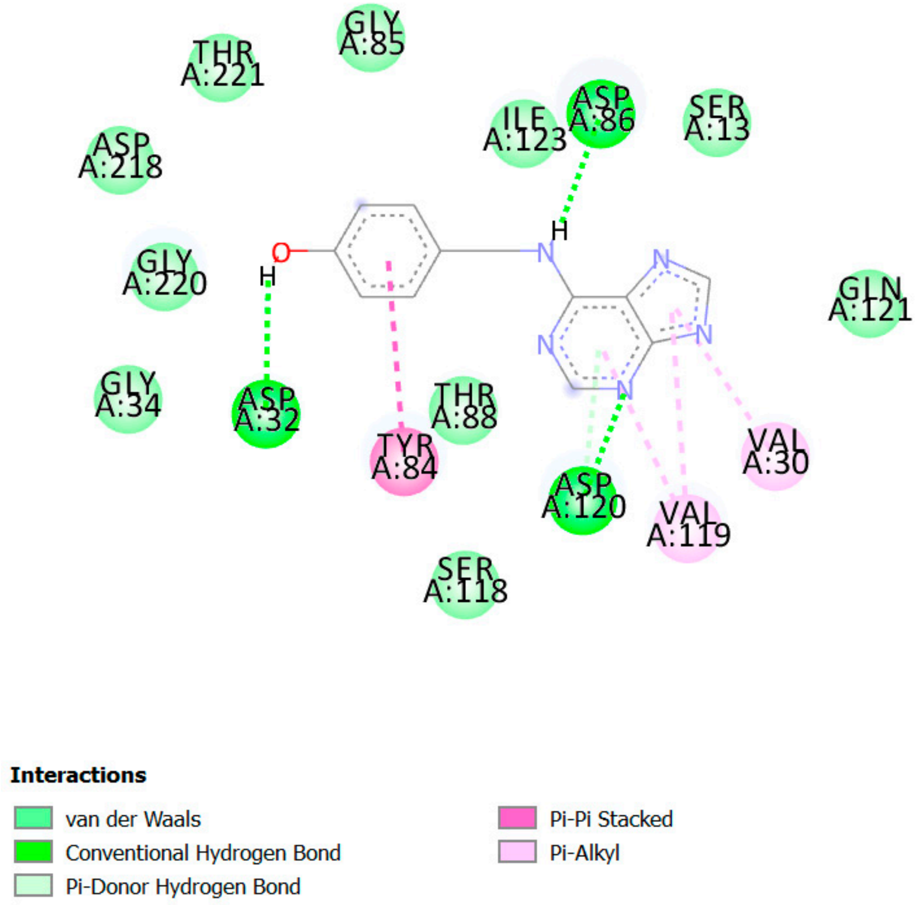

Figure S7. Two-dimensional representation of interactions between molecule *p*-**T** and amino acids from SAP active site.

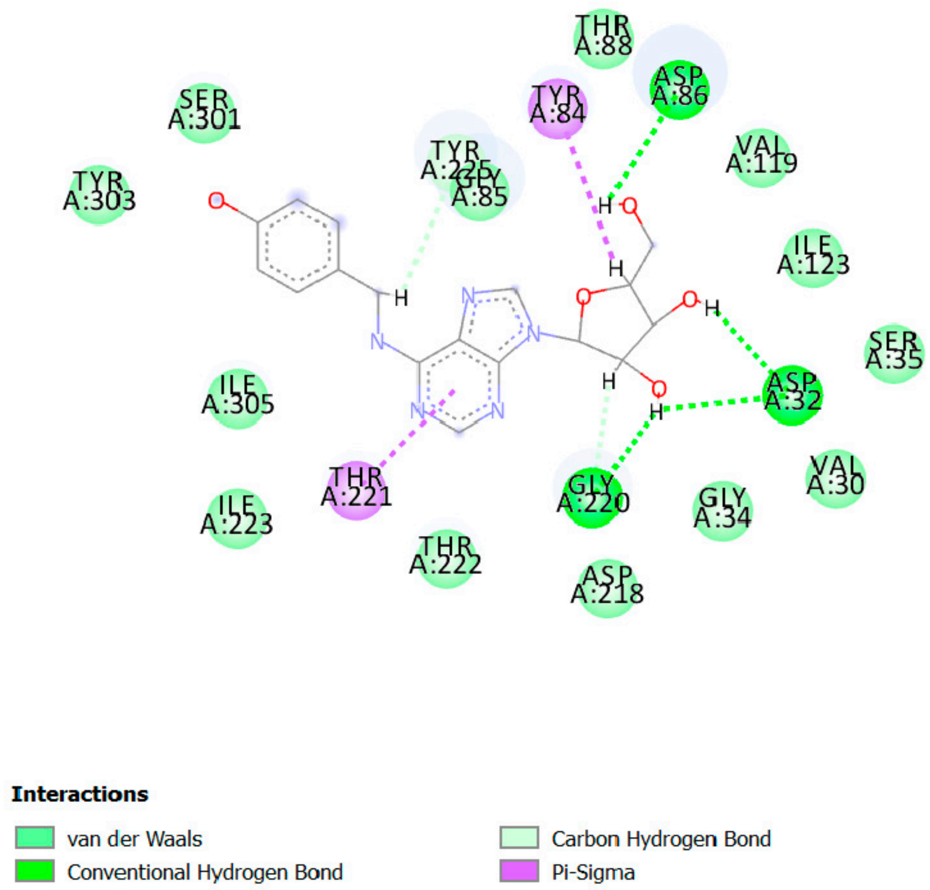

Figure S8. Two-dimensional representation of interactions between molecule *p*-**TR** and amino acids from SAP active site.

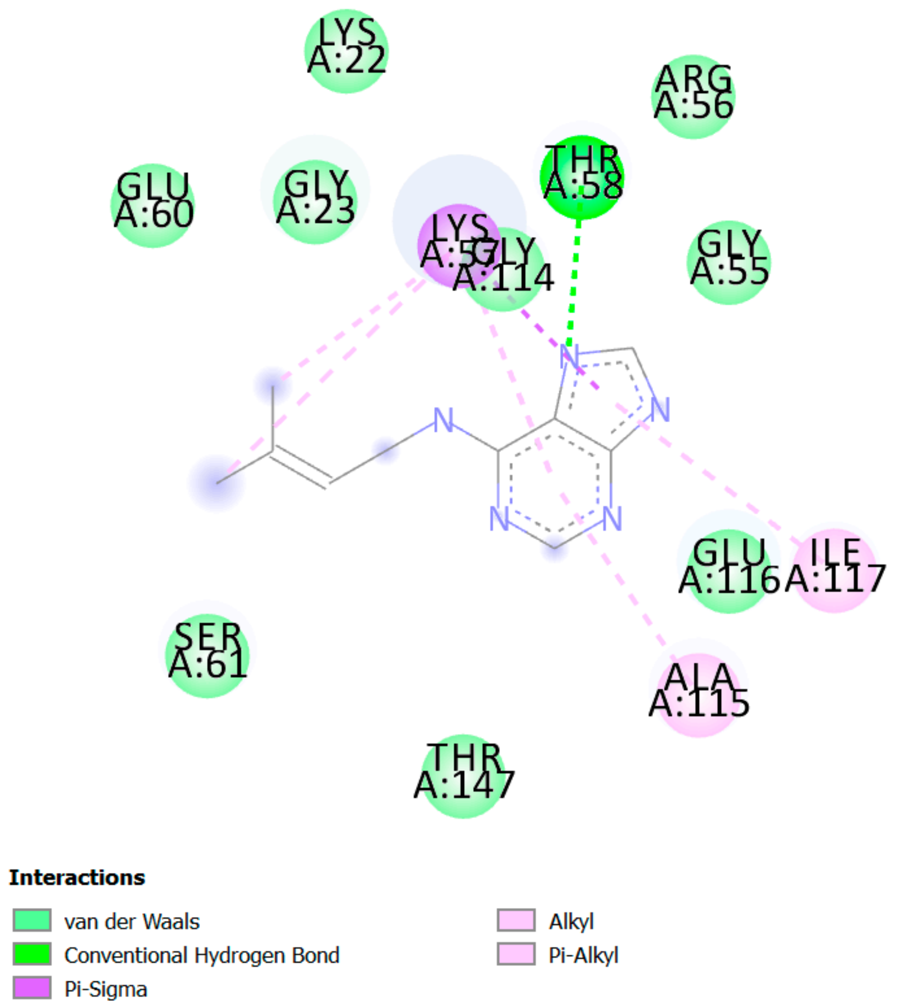

Figure S9. Two-dimensional representation of interactions between molecule **iPA** and amino acids from DHFR active site.

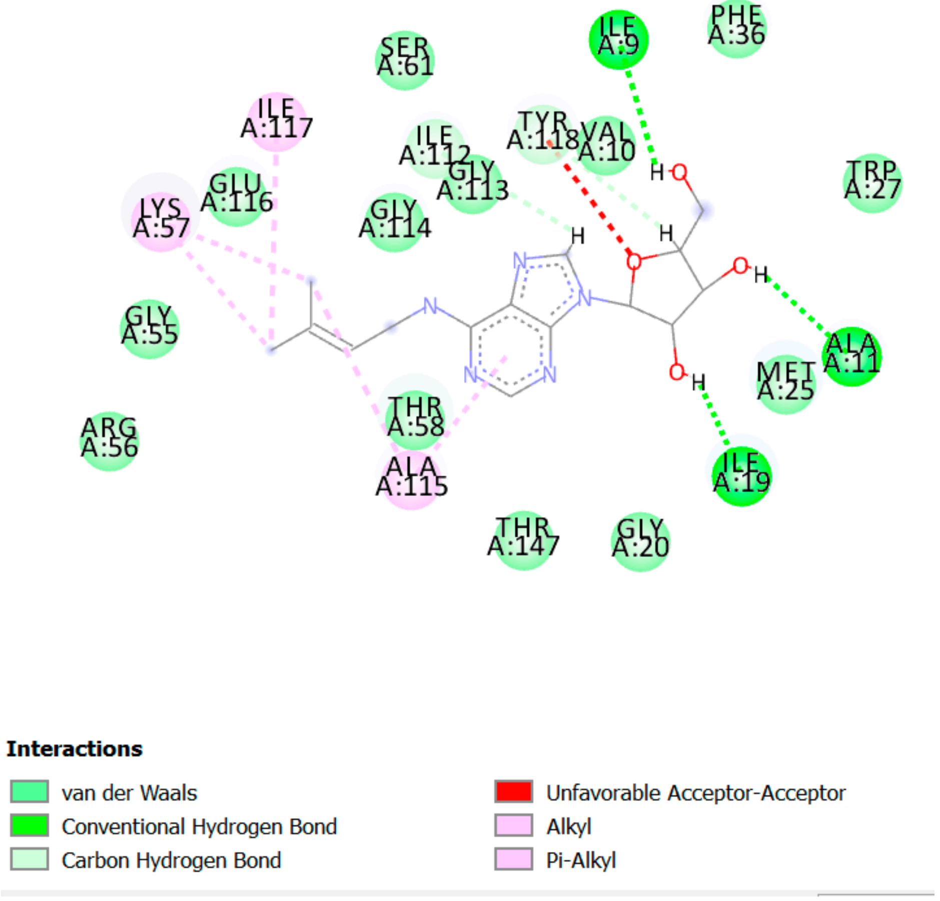

Figure S10. Two-dimensional representation of interactions between molecule **iPAR** and amino acids from DHFR active site.

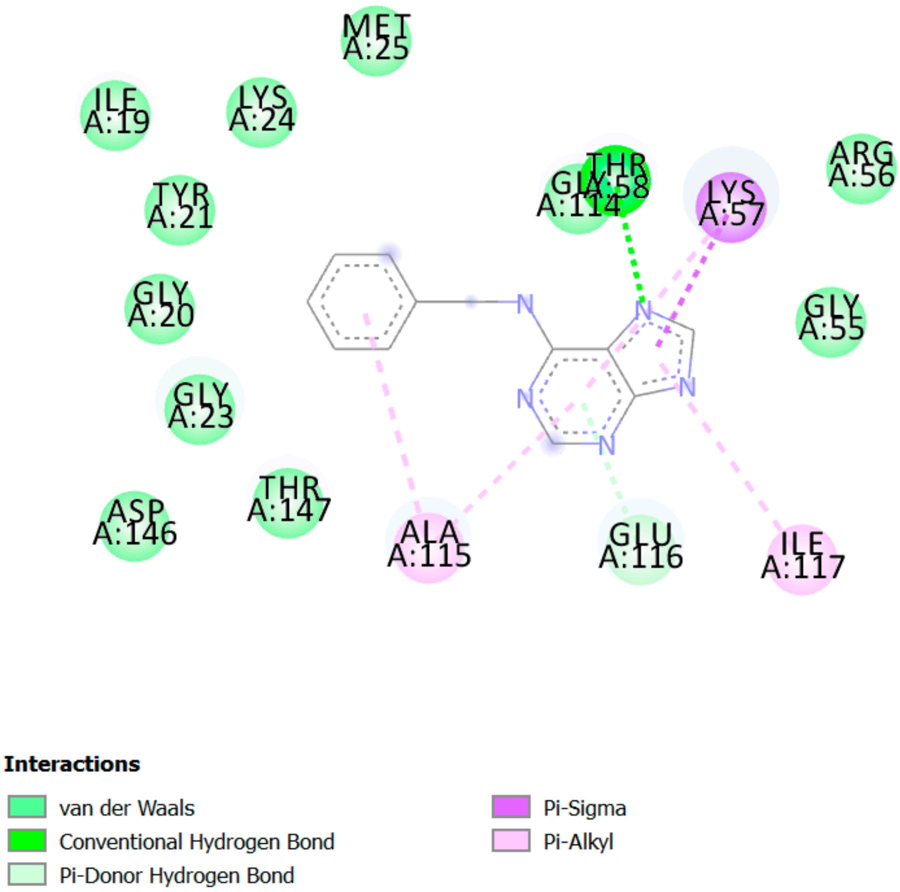

Figure S11. Two-dimensional representation of interactions between molecule **B** and amino acids from DHFR active site.

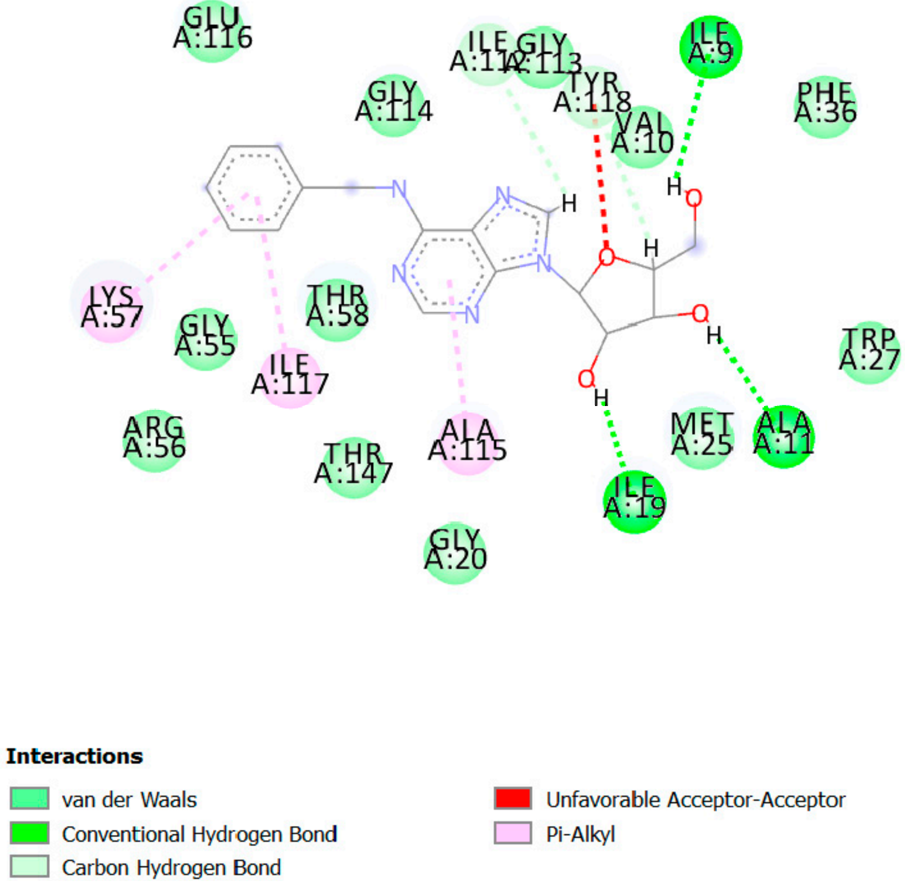

Figure S12. Two-dimensional representation of interactions between molecule **BR** and amino acids from DHFR active site.

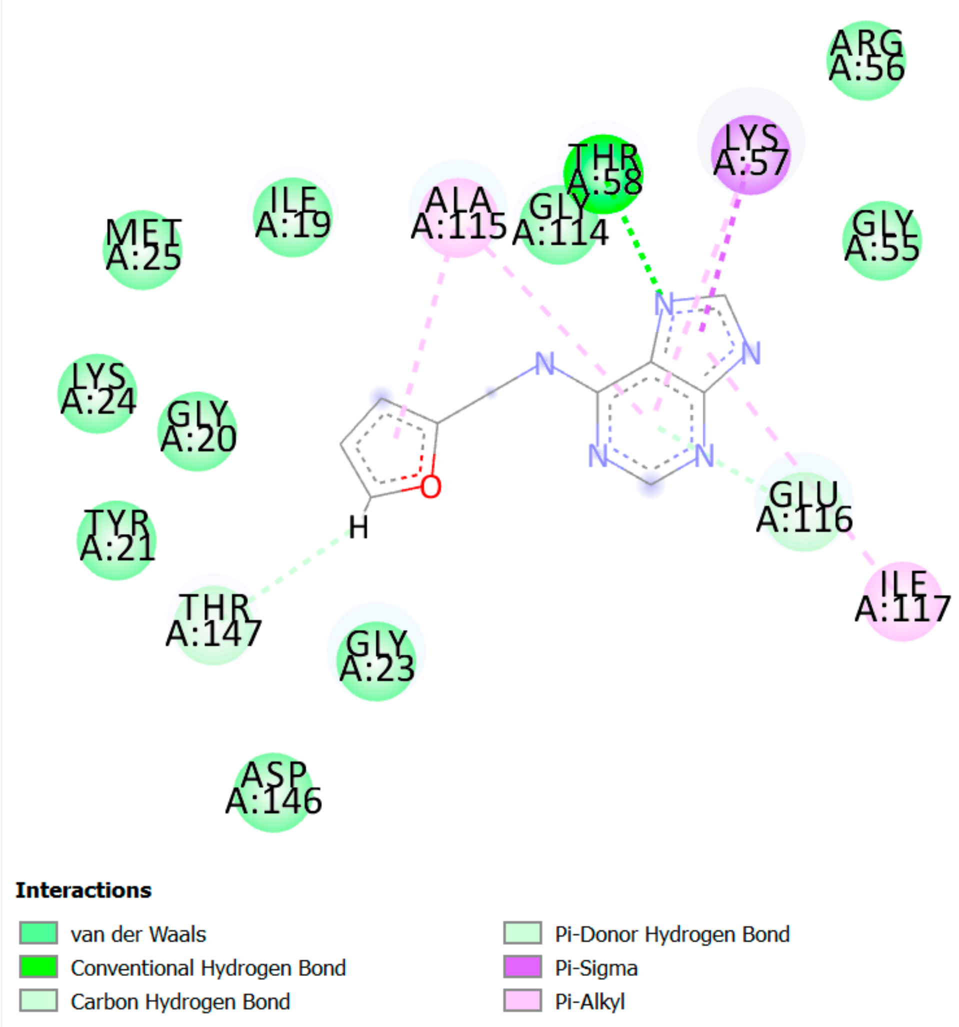

Figure S13. Two-dimensional representation of interactions between molecule **K** and amino acids from DHFR active site.

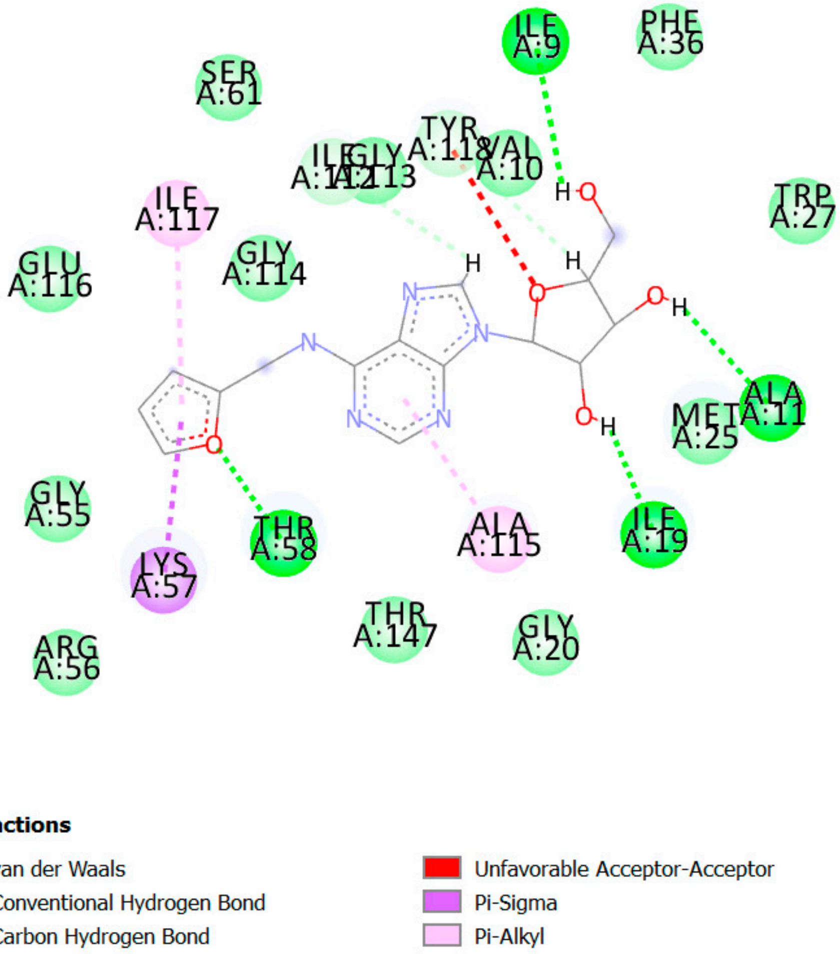

Figure S14. Two-dimensional representation of interactions between molecule **KR** and amino acids from DHFR active site.

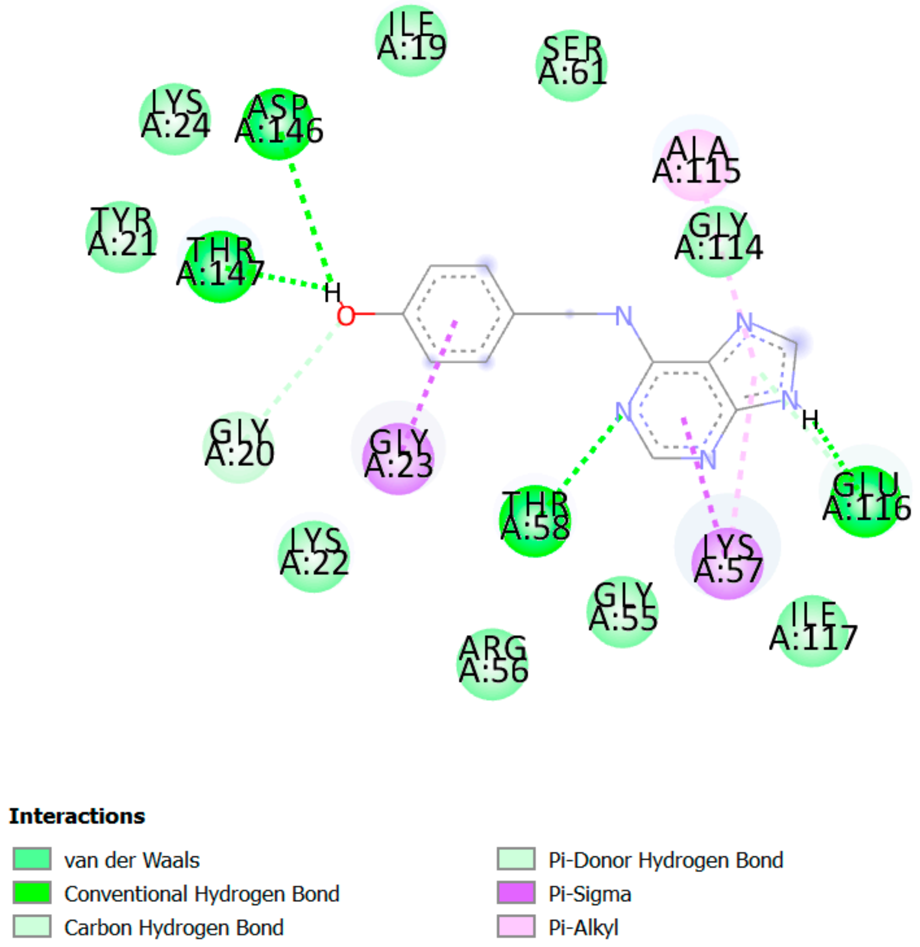

Figure S15. Two-dimensional representation of interactions between molecule *p*-**T** and amino acids from DHFR active site.

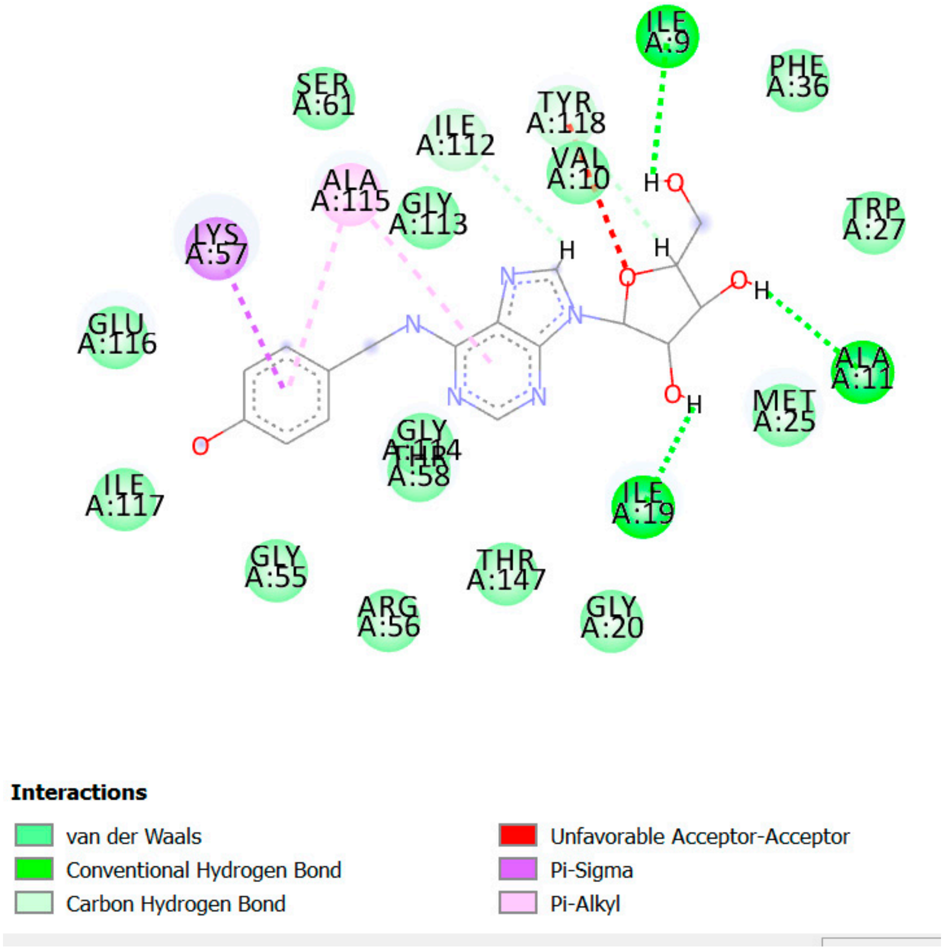

Figure S16. Two-dimensional representation of interactions between molecule *p*-**TR** and amino acids from DHFR active site.
